# From theory to compassion: Why duloxetine matters for chemotherapy-induced neuropathy in the Global South

**DOI:** 10.1017/S1478951525101004

**Published:** 2025-10-29

**Authors:** Jose Eric Mella Lacsa

**Affiliations:** Theology and Religious Education, De La Salle University, Manila, Philippines

Dear Editor,

I read with interest the recent article by Mastroleo et al. (2025) which concludes that despite theoretical concerns about duloxetine reducing tamoxifen efficacy, no clinically significant interaction has been demonstrated, and duloxetine should remain an option for managing chemotherapy-induced peripheral neuropathy in estrogen receptor–positive breast cancer. This is a timely reminder of how evidence and patient experience often meet at the bedside. Chemotherapy-induced peripheral neuropathy (CIPN) continues to be one of the most difficult symptoms faced by cancer patients and survivors, robbing them of mobility, function, and comfort. Among available drugs, duloxetine stands out as the only treatment consistently shown to bring relief, yet its use has long been clouded by concern over a possible interaction with tamoxifen.

What the literature increasingly shows is that this concern remains theoretical rather than real. Mastroleo and colleagues highlight a case that brings this home, patients who find meaningful relief with duloxetine should not be denied that chance because of an unproven risk. For those of us working in resource-limited settings like the Philippines, this is not a minor debate. It touches directly on access, equity, and the everyday struggle of patients who already face limited drug choices and fragmented cancer care (Fraile-Martínez et al. 2025).

In the Philippine context, many patients pay out-of-pocket for medications, and alternatives such as venlafaxine are not always available, or simply do not work (Lambojon et al. 2020). To withhold duloxetine under these circumstances, when evidence shows no significant compromise of tamoxifen efficacy, risks prolonging suffering that could otherwise be eased. Here, the question becomes not just pharmacologic but ethical: what does it mean to choose caution over compassion when the data no longer support such hesitation?

Globally, the implications are no less urgent. If duloxetine continues to be sidelined, patients, particularly in low- and middle-income countries, stand to lose the only proven agent for CIPN (Wickham 2013). On the other hand, reaffirming duloxetine’s safety and effectiveness can influence guidelines, drug procurement, and clinical practice in ways that extend real relief to patients who need it most.

Cancer care never happens in isolation. It is shaped by health systems, drug supply chains, and the difficult decisions families must make every day (Wang et al. 2024). The duloxetine–tamoxifen debate asks us, as a global palliative care community, to reflect on how we balance theoretical risks against the lived reality of suffering. For patients in places like the Philippines, embracing duloxetine not as a last resort but as a rightful option could make an immeasurable difference.

At its heart, this is a matter of dignity. Pain control is not a luxury; it is a basic need. Duloxetine, reconsidered with both evidence and compassion, points us toward a more humane standard of care – one that resists fear, respects context, and insists that the relief of suffering remains our highest calling.
